# Homophobic Bullying, Traditional Bullying, Cyberbullying, and Health-Related Quality of Life (HRQoL) in Adolescents According to Their Sexual Orientation

**DOI:** 10.3390/bs14080729

**Published:** 2024-08-22

**Authors:** Almudena Hurtado-Mellado, Antonio J. Rodríguez-Hidalgo

**Affiliations:** Department of Psychology, University of Cordoba, 14071 Cordoba, Spain; ahurtado@uco.es

**Keywords:** homophobic bullying, sexual orientation, bullying, cyberbullying, health-related quality of life (HRQoL), discrimination

## Abstract

Recent studies suggest that traditional bullying, cyberbullying, and homophobic bullying lead to a low health-related quality of life (HRQoL) in adolescents. The present study aims to analyze this, paying particular attention to homophobic bullying, based on the sexual orientation of 815 adolescents who were asked to fill in a self-report questionnaire. In heterosexuals, both homophobic victimization and victimization were inversely related to different dimensions of HRQoL (moods and emotions, and school environment, respectively). In adolescents who were uncertain about their sexual orientation, there were inverse relationships between homophobic victimization and mood and social acceptance, and there was also a positive relationship between homophobic aggression and physical well-being. In homosexuals and bisexuals, homophobic victimization was inversely related to social acceptance, economic resources, and autonomy, while homophobic aggression was not related to HRQoL. The results obtained offer new insights, thanks to the use of a specific and validated instrument to record homophobic bullying that covers both homophobic victimization and homophobic aggression. In addition, the need to intervene in a holistic manner, involving political and social agents, as well as all actors implicated in the school environment, is discussed with a view to protecting adolescent health and promoting inclusive education.

## 1. Introduction

The transition from childhood to adolescence implies the opening and adaptation to new social scenarios, through which new relationships are experienced, new roles are assumed, and new competences are deployed [1]. The relationship with peers—which mostly takes place in contexts of school and extracurricular activities—is beginning to be the social scenario that most occupies and worries those who are going through this stage. Being part of peer networks and developing a balanced and healthy personal and social identity are processes that can be affected by the phenomena of violence and/or exclusion among peers [2]. Bullying and cyberbullying seriously harm adolescents’ health and psychosocial development [3]. Their effects can last into adulthood, and this affects both the victims, who experience worse social and psychological health [4,5], and their aggressors, who suffer antisocial and physical health problems [6].

Adolescence is a stage in which teenagers explore their gender and sexual identity [7], and any young people who stand out from the rest of the group for any reason run a greater risk of being bullied by their peers [8,9]. Adolescents can be victims of homophobic bullying [10,11], and this phenomenon predicts different psychological disorders in adolescents [12,13].

Studying the relationships between traditional bullying, cyberbullying, homophobic bullying, and health-related quality of life (hereafter, HRQoL) can help us recognize the key elements needed to improve the prevention and alleviation of discriminatory bullying caused by homophobia. This would make a major contribution in the progress toward achieving the Sustainable Development Goal of the United Nations General Assembly (2021) of safe, non-violent, inclusive schools which facilitate equal learning opportunities for all.

### 1.1. Traditional Bullying, Homophobic Bullying, and Cyberbullying

Bullying occurs when a student is repeatedly harassed, intentionally and with an imbalance of power [14]. This harassment has two interrelated dimensions: aggression and victimization [15]. The generic phenomenon is also known as traditional bullying [16].

Homophobic bullying occurs when the aggression is based on the victim’s real or perceived sexual orientation or gender identity, and the victim is an individual positioned outside the heteronormativity of the group, regardless of whether they belong to the LGBT community or not [17,18]. Homophobic bullying differs from the rest in its origin, prevalence, and consequences. This harassment is caused by social stigmas that encourage the punishment of anyone who breaks the rules established by the group, regardless of their real sexual orientation or gender identity [18]. This prejudice-based harassment has been highlighted in previous research as extremely relevant, among other causes, because it severely affects minority groups [19].

Likewise, the prevalence rates of homophobic bullying range from 22% to 87%, exceeding those of traditional bullying and cyberbullying [17,20]. In addition to finding higher numbers, victims of this phenomenon seem to have a greater difficulty in disclosing what is happening to them as such disclosure can become a source of harassment due to the recognition of a non-heterosexual sexual orientation [21], something that can even lead to the loss of support from both their peers and their reference adults due to social prejudice [22].

When this harassment is carried out via Information and Communication Technologies, it is termed cyberbullying [23,24]. Cyberbullying differs from the previous types of bullying, among other reasons, due to the characteristics of cyberspace: there are no guardians to help minors defend themselves against the attacks received, the capacity to disseminate harmful content is almost unlimited, and the possibility of deleting and completely forgetting it is very limited [25]. Several studies have shown that many more LGBT students end up being victims of cyberbullying compared to their non-LGBT peers (see, e.g., [26]).

Some researchers suggest that traditional bullying could act as a precursor to homophobic taunting (see, e.g., [27]). Participating as an aggressor in traditional bullying could be linked to future involvement as an aggressor in other forms of aggression, including those with homophobic overtones [28,29].

In addition, there seems to be an overlap between homophobic bullying and cyberbullying, especially when non-heterosexual students are the victims [30]. Furthermore, LGBT students are at a greater risk of polyvictimization [31].

Despite the increasing number of studies on the relationship between homophobic bullying and traditional bullying or cyberbullying, there is still a lot we do not know about it. This review of the literature will therefore also cover the relationship between traditional bullying and cyberbullying. These two forms of bullying seem to occur simultaneously in adolescents [32,33] and appear to be related to each other.

In addition, it is important to stress that a relationship exists not only between the two phenomena, but also between the different forms of involvement in them: in particular, between aggression and victimization, both within the same form of bullying and between them. In this context, the studies on homophobic bullying are scarcer and focus mostly on victimization [8,34]. For this reason and in order to obtain more information, we expanded the search to include information from research which focuses on traditional bullying and cyberbullying. Aggression and victimization are directly related to each other [35], and being a victim of traditional bullying could also predict a person’s involvement as an aggressor. There is also disagreement over how these roles change when they are involved in more than one type of bullying. On the one hand, some studies show that a student involved in traditional bullying will play the same role when they move to online bullying [23,36], while other research concludes that the roles can vary and combine when the type of bullying changes [32,37].

### 1.2. Traditional Bullying, Homophobic Bullying, Cyberbullying, and Health-Related Quality of Life

Traditional bullying, homophobic bullying, and cyberbullying all have a negative psychological and social impact on young people [38,39,40]. These forms of violence affect all those involved by lowering their HRQoL, which is defined as the perception that a person holds about their physical, psychological, and social condition [41,42]. Several studies have shown that bullying and HRQoL are related in two ways: (a) bullying affects the quality of life of all those involved; and (b) the fall in HRQoL leads to increased aggressiveness [43,44].

A growing body of research from different countries shows how being a victim of bullying at school is positively associated with a state of emotional instability, lower self-esteem, low energy, low vitality, limitations in physical activities, lower psychological well-being, symptoms of depression, and in general, a lower HRQoL than that of students who are not bullied [2,41,45]. In a study carried out in Norway, threats and bullying predicted a low HRQoL [46]. Along these lines, recent studies have shown that students who have not been involved in any type of harassment obtain better scores on quality of life markers [47,48].

In both the victims and the aggressors of bullying, different dimensions of HRQoL are affected. In a study conducted in 11 European countries in situations of bullying, HRQoL was significantly worse in all dimensions measured with KIDSCREEN-52 [49]. The strongest association was between bullying and unhappy moods and emotions, while the weakest link was between bullying and low physical well-being. According to Haraldstad et al. [44], victims of bullying were generally unhappier, had a more negative self-perception and a worse relationship with their school, and complained more about their families’ financial resources. As for the impact on the aggressors’ HRQoL, bullying seems to have a very strong negative association with the dimension of the school environment.

One of the most recent studies about the relationship between cyberbullying and HRQoL, by González-Cabrera et al. [50], shows that the dimensions which are negatively related to both cybervictimization and cyberbullying are those of mood and emotions, psychological well-being, parental relationships and family life, school environment, and social rejection.

However, research into the relationship between homophobic bullying and HRQoL is much less common. These studies have focused more on HRQoL in victims of homophobic bullying. Homophobic victimization has been described as damaging the physical and mental health of those affected [51]. Victims of homophobic bullying tend to show higher levels of depression and anxiety and a worse physical condition than the rest of their non-bullied peers (see, e.g., [52,53]). To our knowledge, no study to date has explored the relationship between homophobic aggression and HRQoL, so this subject is largely uncharted territory. Given the scarcity of studies on homophobic bullying and HRQoL, more research certainly needs to be conducted in this area.

### 1.3. The Present Study

According to the scientific literature, traditional bullying, homophobic bullying, and cyberbullying in adolescence threaten and seriously harm quality of life. In this line of research, studies seem to indicate that LGBT adolescents suffer more from these forms of violence among peers and tend to have a lower HRQoL.

Furthermore, most of the studies which focus on homophobic bullying use questionnaires or self-report scales on traditional bullying as measurement instruments, as well as utilizing sociodemographic variables such as sexual orientation and/or gender identity. Although this has led to increased awareness about the vulnerability of LGBT minors to bullying, these kinds of studies present some inaccuracies: when generic bullying instruments are used, incidents of victimization which are not specifically homophobic are also included; all of the bullying suffered by people who recognize themselves as LGBT tends to be labeled as homophobic in nature; the fact that homophobic bullying can be suffered by people who do not identify themselves as LGBT is ignored; and finally, the focus is mainly on victimization, with little attention paid to homophobic aggression.

The main purpose of the present study is to explore the relationships between homophobic bullying (homophobic aggression and homophobic victimization) and traditional bullying (aggression and victimization), cyberbullying (cyberbullying and cybervictimization), and HRQoL, based on the sexual orientation of adolescents (homosexual or bisexual, those who are unsure about their sexual orientation, and heterosexual). The study hypotheses are as follows:

(1) There will be positive relationships between the levels of aggression and/or victimization in homophobic bullying, traditional bullying, and cyberbullying.
(2) The levels of homophobic victimization will be inversely related to the levels of HRQoL, especially in the dimensions of moods and emotions, social support and peers, financial resources, and psychological well-being.
(3) The levels of homophobic aggression will be inversely related to the levels of HRQoL, particularly in the dimension of the school environment.
(4) The patterns of relationships between the variables will differ depending on the sexual orientation of the group studied—homosexuals and bisexuals, individuals unsure of their sexual orientation, and heterosexuals.

## 2. Materials and Methods

### 2.1. Participants

This study was performed with a population of school students ranging from the 1st year of compulsory Secondary Education, or ‘E.S.O.’, to the 2nd year of High School in state-run schools in Córdoba (Spain). Using intentional accessibility sampling, a sample of 815 participants was obtained, of which 51.6% were girls and 48.4% boys. Their ages ranged from 11 to 19 years (Age M = 14.87; SD = 1.72) and there was a variety in terms of sexual orientation: 82.1% considered themselves heterosexual (n = 669), 6% homosexual or bisexual (n = 49), and 11.9% said they were unsure of their sexual orientation (n = 97).

### 2.2. Procedure

A total of five educational institutions participated with the following inclusion criterion: secondary schools in the province of Córdoba. First, the schools were contacted to request their voluntary participation in the study. After this was given, we asked for authorization from the students’ parents or legal guardians. Next, the students were given a brief explanation about the study, in which they were informed that participation was voluntary and anonymous, and that the confidentiality of the data they provided would be guaranteed. The researchers then explained the different instruments, and in addition, a brief description of each instrument was included in the questionnaires. After this, a self-report battery was administered to each student in person in a 40–60 min session, depending on the age of the group, during school hours. The questionnaire was answered on paper by the students, who completed it in their usual classroom, in the same space as their classmates (groups of between 15 and 25 participants) but at separate tables, so that they could complete it freely and individually. In each classroom, there was a researcher who explained the procedure and answered any questions that may have arisen. The research team respected the ethical principles of the Declaration of Helsinki of the World Medical Association and the national laws regulating research and psychologists’ professional conduct. The procedure was also approved by the Ethics Committee of the University.

### 2.3. Instruments

To carry out this study, we used a self-report battery composed of the following questionnaires:

***Sociodemographic data:*** questions referring to school year, gender, age, and sexual orientation, among others. As sexual orientation and gender identity are important constructs in this study, the questionnaire provided several alternatives for young people to freely express their identity, including an open-response option in case the preset options did not fit their identification.

***Sexual orientation:*** For this section, we followed the model from Poteat, Espelage, and Koenig [54], using the question “Do you feel confused about being homosexual or bisexual?” The answers to choose from were as follows: (1) Never confused, because I know I am heterosexual; (2) Rarely confused; (3) Sometimes confused; (4) Fairly confused; or (5) Never confused, because I know I am homosexual or bisexual.

***Bullying:*** We used the Spanish version [36] of the European Bullying Intervention Project Questionnaire (EBIP-Q) [55]. This instrument is made up of 14 items, of which 7 are used to measure victimization (α = 0.751) and 7 are for aggression (α = 0.780). The questions were answered based on a 5-point Likert scale.

***Homophobic bullying:*** We used an adapted Spanish version [36] of the European Bullying Intervention Project Questionnaire (EBIP-Q) [55]. This adaptation has a different explanatory introduction to guide the participants’ responses. In the original questionnaire, the response is oriented to the bullying situations experienced, without specifying the motivation. The introduction was changed so that the answers referred only to situations of homophobic bullying: “The next question is about your possible experiences of discrimination in your own environment (school, friends, acquaintances) against heterosexuals, homosexuals or bisexuals, due to their differences in sexual orientation or gender identity, over the last two months”. The introduction was followed by the 14 questions from the original instrument, 7 of which measured victimization (α = 0.855) and 7 measured aggression (α = 0.780). The questions were measured on a 5-point Likert scale with 1 = “No” and 5 = “Yes, more than once a week”.

***Cyberbullying:*** This was measured using the Spanish version of the European Cyberbullying Intervention Project Questionnaire (ECIPQ) [56]. It includes 22 items divided into two dimensions, with 11 questions referring to cybervictimization (α = 0.820) and 11 referring to cyberaggression (α = 0.835). A Likert-type scale between 1 and 5 was used for the answers, with 1 = “No” and 5 = “Yes, more than once a week”.

***Health-related quality of life:*** We used the KIDSCREEN-52 questionnaire to measure the participants’ HRQoL [57]. This questionnaire contains 52 items which measure 10 dimensions: 5 items (α = 0.868) are about physical well-being, 6 items (α = 0.917) about psychological well-being, 7 items (α = 0.902) about mood, 5 items (α = 0.265) about self-perception, 5 items (α = 0.898) about autonomy, 6 items (α = 0.894) about the relationship with parents and family life, 6 items (α = 0.830) about friends and social support, 6 items (α = 0.842) about the school environment, 3 items (α = 0.762) about social acceptance, and 3 items (α = 0.862) about money matters. A 5-point Likert response scale is used to answer the questions, with 1 = “Never”/“Not at all” and 5 = “Always”/“Extremely”.

### 2.4. Analytic Strategy

Bivariate correlation analyses were carried out to study the relationships found between homophobic victimization and aggression and the other study variables. The variables introduced in the analyses were as follows: homophobic bullying victimization (HV), homophobic bullying aggression (HA), physical well-being (PhW), psychological well-being (PsW), moods and emotions (MEs), self-perception (SelfP), autonomy (Auto), parental relations and home life (PrHl), financial resources (FRs), social support and peers (SsP), school environment (SE), social acceptance (SA), traditional bullying victimization (TBV), traditional bullying aggression (TBA), cybervictimization (CBV), and cyberaggression (CBA).

We followed this procedure for each of the groups—heterosexuals, those who were unsure about their sexual orientation, and homosexuals or bisexuals—to find out the differences in the relationships between the variables based on the sexual orientation of those involved. The results for each group are presented below in Table 1.

## 3. Results

### 3.1. Group with Heterosexual Sexual Orientation

The results in the group of adolescents who consider themselves heterosexual showed that homophobic bullying victimization had the highest significant correlations with homophobic bullying aggression (r = 0.401, *p* < 0.01), as well as with other forms of victimization such as cybervictimization (r = 0.255, *p* < 0.01) and traditional bullying victimization (r = 0.246, *p* < 0.01). It also correlated positively with cyberaggression and traditional bullying aggression, albeit to a lesser extent. On the other hand, significant negative correlations were found with the dimensions of moods and emotions, financial resources, and social acceptance (see Table 1).

As regards homophobic bullying aggression, the most significant correlations in this group, in addition to those for homophobic bullying victimization, were found with the variables of cyberaggression (r = 0.406, *p* < 0.01) and traditional bullying aggression (r = 0.428, *p* < 0.01). In line with what happens in victimization, homophobic bullying aggression shows a significant positive relationship with cybervictimization (r = 0.325, *p* < 0.01) and traditional bullying victimization (r = 0.244, *p* < 0.01). In addition, the results point to inverse correlations with various dimensions of HRQoL such as moods and emotions (r = 0.096, *p* < 0.05), parental relations and home life (r = 0.148, *p* < 0.01), financial resources (r = 0.114, *p* < 0.01), and school environment (r = 0.228, *p* < 0.01).

### 3.2. Group Unsure of Their Sexual Orientation

As for the group of adolescents who are unsure about their sexual orientation, the analyses suggest that homophobic bullying victimization correlates positively and significantly with homophobic bullying aggression (r = 0.428, *p* < 0.01), as well as with the other dimensions related to both traditional bullying and cyberbullying, except for traditional bullying aggression, where no significant relationship was found (see Table 1). Regarding the dimensions of HRQoL, homophobic bullying victimization correlated negatively with moods and emotions (r = 0.212, *p* < 0.05) and social acceptance (r = 0.341, *p* < 0.01).

On the other hand, homophobic bullying aggression, in addition to homophobic bullying victimization, was significantly related to the two dimensions of cyberbullying and traditional bullying aggression, as shown in Table 1. Similarly, in this group, homophobic bullying aggression correlated positively with physical well-being (r = 0.239, *p* < 0.05) and negatively with social acceptance (r = −0.268, *p* < 0.01).

### 3.3. Group with Homosexual or Bisexual Sexual Orientation

In the group of young people who stated they are homosexual or bisexual, the results show that homophobic bullying victimization and homophobic bullying aggression were not significantly correlated. Homophobic bullying victimization was positively related to the other two forms of victimization studied (see Table 1). It was also related, but inversely, to the dimensions of autonomy (r = −0.286, *p* < 0.05), financial resources (r = −0.453, *p* < 0.01), and social acceptance (r = −0.357, *p* < 0.05).

However, in this group, homophobic bullying aggression only correlated significantly with cyberbullying, in both dimensions of cyberaggression and cybervictimization. There were no significant relationships with the other variables.

## 4. Discussion

The first hypothesis suggested that there would be positive relationships between the levels of aggression and/or victimization in homophobic bullying, traditional bullying, and cyberbullying. This hypothesis was largely corroborated in each of the groups based on their sexual orientation. In the heterosexual group, the hypothesis was corroborated in all of its terms, which is consistent with the observations made by other studies (see, e.g., [35]). However, these studies do not draw any conclusions about which one causes the other. It may be a case of a chain effect: being involved in some type of bullying and/or cyberbullying could lead young heterosexual people to tend toward other forms of aggression and victimization such as homophobic bullying, and vice versa. Heterosexuals who are victims of homophobic aggression could react by being aggressive to defend themselves against these attacks and to try to use their own aggression to reinforce the status of their sexual orientation [7]. This would generate rejection from the group and could lead to other forms of aggression and victimization, and the relationship would end up in a vicious circle.

In the group of participants who are unsure of their sexual orientation, we see a very similar pattern emerging in relation to the first hypothesis. However, there is no relationship between homophobic victimization and bullying aggression, or between homophobic aggression and bullying victimization. This is consistent with studies which show that the roles are maintained between different forms of bullying [23]. However, relationships have also been observed between the levels of homophobic victimization and those of homophobic aggression and cyberaggression, as well as between those of homophobic aggression and those of homophobic victimization and cybervictimization. Given that we are referring to the group of young people who are unsure about their sexual orientation, one possible explanation could be that these adolescents take part in homophobic aggression to assert their conformity with established rules of gender and sexuality in front of the group [58]. Such aggression could result in a similar response on the part of their victims or other schoolmates, thus establishing a vicious circle between aggression and victimization. The change of roles when perpetrating cyberbullying, however, could be due to the new scenario offered by the online medium, in which the anonymity affords victims enough safety to respond to the attacks [32].

The group in which the first hypothesis was least evidenced was that of the students with homosexual or bisexual orientation. In their case, homophobic victimization was not related to homophobic, traditional, or online bullying. It could be inferred that homophobic victims in this group show less aggressive response behavior toward their peers compared to their classmates. In homosexuals and bisexuals, high levels of homophobic victimization are associated with high levels of victimization by bullying and cybervictimization. This is consistent with maintaining the role of a victim through different types of bullying [36] and with theories which point to the existence of polyvictimization in LGBT adolescents [30]. LGBT students who are victims of bullying may become the target of other types of bullying and, in turn, may not feel capable of responding to it, because they receive attacks more frequently [18] and the consequences for them are more serious [59]. Homophobic aggression perpetrated by homosexuals and bisexuals is positively related to both cyberaggression and cybervictimization, which agrees with the findings of the study by Hemphill et al. [60].

The scientific literature we reviewed points to the existence of an inverse relationship between involvement in any form of bullying and HRQoL [50]. High levels of victimization are also associated with low levels of HRQoL and vice versa [41,45]. Our initial hypothesis was that there would be an inverse relationship between homophobic victimization and HRQoL, especially in its dimensions of moods and emotions, social support and peers, financial resources, and psychological well-being. This was partially corroborated, with certain differences found according to the sexual orientation of the participants. In heterosexuals, homophobic victimization was inversely related to the dimensions of moods and emotions, financial resources, and social acceptance. There is no precedent for such a conclusion in the scientific literature, since homophobic victimization has always been studied preferentially from the viewpoint of the LGTB group and not with respect to heterosexuals. The negative relationship detected between social acceptance and homophobic victimization seems to concur with the conclusions of some studies which suggest that group support can act as a protective factor [61]. It would therefore be logical to think that acceptance by the group could be related to low victimization, or vice versa, although it could also be that the group rejects the victim in order not to become targets of the aggressors themselves. On the other hand, the inverse relationship of homophobic victimization in heterosexual adolescents with mood and financial resources coincides with the findings of some previous research [59].

In adolescents who are unsure about their sexual orientation, there was an inverse relationship between the levels of homophobic victimization and those of moods and social acceptance. This coincides with the previous literature, which points to social acceptance as a protective factor [62]. We have not found any studies in the literature which focus specifically on adolescents with an undefined sexual orientation, so these results could help us to begin to grasp how these two phenomena are related in this group. The dimensions of HRQoL are related to homophobic victimization in a very similar way to that of the group of heterosexual participants, perhaps due to the fact that most adolescents who are unsure of their sexual orientation would be unlikely to reveal these doubts to their peers and would prefer to appear heterosexual in front of their classmates.

In homosexual or bisexual adolescents, the levels of homophobic victimization had an inverse relationship with the levels of social acceptance (also observed in heterosexuals and those unsure of their sexual orientation) and economic resources and autonomy (only observed in this group of adolescents). Although no previous scientific research has been found on these relationships for this group of adolescents, the closest finding was the evidence that autonomy was inversely related to victimization by traditional bullying (see, e.g., [44]). It would therefore be logical to think that in contexts of vulnerability and/or social exclusion, homosexual or bisexual adolescents are at a greater risk of being the target of homophobic victimization. For this group of adolescents, we could also assume that being the object of homophobic victimization could lead to lower confidence and self-esteem, and greater heteronomy. Blais, Gervais, and Hébert [10] concluded in their study that adolescents with a non-heterosexual sexual orientation who suffered homophobic victimization tended to identify with the thoughts of the aggressors and scored higher than them in internalized homophobia. Moreover, rejection by peers—mostly heteronormative—could encourage bullied homosexual or bisexual adolescents to assimilate the predominant ideas of their aggressors’ peer group in order to try to fit in with the group.

The levels of homophobic aggression were expected to be negatively related to HRQoL levels, especially those of the dimension of school environment, in all three groups. However, we were only able to corroborate this hypothesis in heterosexual adolescents. This relationship is found in the previous literature, but only in a study in which the variable of sexual orientation was not taken into account [44]. In addition to this correlation, we found in heterosexual adolescents that homophobic aggression was inversely related to the dimensions of moods, parents and family life, and economic resources. Previous studies suggest that the lower HRQoL may be linked to increased aggressiveness [42]. It therefore seems logical to think that in this group of heterosexual adolescents, insufficient communication with the parents and a troubled family environment, together with low financial resources and a negative mood, could be closely related to an increase in aggressive behavior toward their parents and peers, the latter in a school environment which they consider unfavorable and/or frustrating.

In adolescents who are unsure about their sexual orientation, homophobic aggression is positively related to physical well-being. This is consistent with the previous observations made in the study by Chen and Huang [63]. In addition, homophobic aggression in this group also correlates, although in this case inversely, with social acceptance. To interpret this, it is important to consider that these adolescents do not have a defined sexual orientation, so feeling rejected by the group could lead to increased aggressiveness as a means of self-defense or a response to that rejection. It may also be that, finding themselves in this situation, they use homophobic bullying to avoid being included in a group which is bullied more often [34].

Finally, in the homosexual or bisexual group, no relationship was observed between homophobic aggression and HRQoL.

Since it has been observed in the previous literature that sexual orientation influences how students are affected by bullying [30,64], the fourth and final hypothesis of this study suggested that the correlational patterns would be different according to the sexual orientation of the participants. This hypothesis was confirmed, since, although some relationships coincided in more than one group, the complete pattern was not repeated in any case.

The results obtained for the relationship found between homophobic bullying, traditional bullying, cyberbullying, and HRQoL show a closer coincidence with previous studies for the heterosexual sexual orientation group, perhaps because most of these previous studies focused on traditional bullying and were usually based on samples in which most of the participants were heterosexual, and sexual orientation was not taken into account. For this reason, it is essential to conduct further research along these lines in order to gain broader knowledge of these phenomena while taking into account the role of diversity in young people’s sexual orientation. The present study offers new knowledge, made possible by the use of a specific, validated instrument to record homophobic bullying which encompasses both victimization and homophobic aggression.

## 5. Conclusions

Based on the conclusions of this study, contributions can be made to try to improve the quality of life of these adolescents, to protect their health, and to promote inclusive education, which will have lasting repercussions on their adult lives. Political and/or social agents should raise awareness, social sensitivity, and commitment and should work to transform people’s attitudes and social practices regarding the diversity of sexual orientation through the enactment of inclusive policies that fight for the rights and welfare of the LGBT community and all adolescents, and through socio-educational programs that work along these lines. We propose that schools should include educational activities in their academic curriculum which promote knowledge and recognition of human diversity in the area of sexual orientation. This work should be aimed at eradicating prejudices against homosexuality and bisexuality, both in face-to-face relationships and online.

This study presents certain limitations which could lead to possible future lines of research. Firstly, the sample size was small; in future research, it would be advisable to use larger samples. Secondly, while the cross-sectional design is appropriate for the purpose of the present study, a longitudinal study should be carried out once the existing relationships between the different aspects are known, which would allow us to infer causality between the factors. It is essential that societies generate healthy environments in which the future generations can develop, and which foster acceptance and freedom of choice when it comes to sexual orientation.

## Figures and Tables

**Table 1:** Pearson correlations for each group.

	Heterosexual		Unsure		Homosexual and Bisexual	
	1. HV	2. HA	1. HV	2. HA	1. HV	2. HA
**1. HV**	-		-		-	
**2. HA**	**0.** 401 **	-	**0.** 436 **	-	0.015	-
**3. PhW**	−0.073	−0.050	0.058	**0.** 239 *	−0.084	0.004
**4. PsW**	−0.036	−0.027	−0.082	0.143	−0.113	0.181
**5. ME**	**−** **0.** 116 **	**−** **0.** 096 *	**−** **0.** 212 *	0.046	−0.241	0.122
**6. SelfP**	0.051	0.065	0.094	−0.054	0.100	−0.074
**7. Auto**	−0.022	−0.033	0.071	0.089	**−** **0.** 286 *	−0.094
**8. PrHl**	−0.071	**−** **0.** 148 **	−0.136	0.092	−0.255	0.026
**9. FR**	**−** **0.** 117 **	**−** **0.** 114 **	−0.094	−0.017	**−** **0.** 453 **	−0.128
**10. SsP**	−0.069	−0.071	−0.146	0.006	−0.167	−0.024
**11. SE**	−0.037	**−** **0.** 228 **	−0.170	0.109	−0.099	−0.013
**12. SA**	**−** **0.** 159 **	−0.043	**−** **0.** 341 **	**−** **0.** 268 **	**−** **0.** 357 *	−0.277
**13. TBV**	**0.** 246 **	**0.** 244 **	**0.** 465 **	0.148	**0.** 599 **	0.164
**14. TBA**	**0.** 183 **	**0.** 428 **	0.151	**0.** 245 *	−0.193	0.223
**15. CBV**	**0.** 255 **	**0.** 325 **	**0.** 336 **	**0.** 498 **	**0.** 427 **	**0.** 613 **
**16. CBA**	**0.** 199 **	**0.** 406 **	**0.** 326 **	**0.** 540 **	0.114	**0.** 614 **

HV: homophobic bullying victimization; HA: homophobic bullying aggression; PhW: physical well-being; PsW: psychological well-being; ME: mood and emotion, SelfP: self-perception; Auto: autonomy; PrHl: parental relations and home life; FR: financial resource; SsP: social support and peers; SE: school environment; SA: social acceptance; TBV: traditional bullying victimization; TBA: traditional bullying aggression; CBV: cybervictimization; CBA: cyberaggression. * *p* < 0.05; ** *p* < 0.01. Significant values have been highlighted in bold.

## Data Availability

Data are contained within the article.

## References

[1] Benítez F.J., Rodríguez-Hidalgo A.J., Herrera-López M. (2023). Propiedades Psicométricas Del Cuestionario Habilidades Socio-Emocionales Para La Mediación Escolar. Electron. J. Res. Educ. Psychol..

[2] Benítez F.J., Rodríguez-Hidalgo A.J., Herrera-López M. (2024). La Competencia Social Multidimensional En La Formación de Las Habilidades Socioemocionales Para La Mediación Escolar. Electron. J. Res. Educ. Psychol..

[3] Martínez J., Rodríguez-Hidalgo A.J., Zych I. (2020). Bullying and Cyberbullying in Adolescents from Disadvantaged Areas: Validation of Questionnaires; Prevalence Rates; and Relationship to Self-Esteem, Empathy and Social Skills. Int. J. Environ. Res. Public Health.

[4] Undheim A.M., Wallander J., Sund A.M. (2016). Coping Strategies and Associations with Depression among 12- to 15-Year-Old Norwegian Adolescents Involved in Bullying. J. Nerv. Ment. Dis..

[5] Wolke D., Copeland W.E., Angold A., Costello E.J. (2013). Impact of Bullying in Childhood on Adult Health, Wealth, Crime and Social Outcomes. Psychol. Sci..

[6] Waasdorp T.E., Pas E.T., Zablotsky B., Bradshaw C.P. (2017). Ten-Year Trends in Bullying and Related Attitudes among 4th- to 12th-Graders. Pediatrics.

[7] Espelage D.L., Basile K.C., Leemis R.W., Hipp T.N., Davis J.P. (2018). Longitudinal Examination of the Bullying-Sexual Violence Pathway across Early to Late Adolescence: Implicating Homophobic Name-Calling. J. Youth Adolesc..

[8] Kahle L. (2020). Are Sexual Minorities More at Risk? Bullying Victimization among Lesbian, Gay, Bisexual, and Questioning Youth. J. Interpers. Violence.

[9] Zych I., Ortega-Ruiz R., Llorent V.J. (2017). Nature and Dynamics of Peer Violence in Polish Upper Secondary Schools. Soc. Psychol. Educ..

[10] Blais M., Gervais J., Hébert M. (2014). Internalized Homophobia as a Partial Mediator between Homophobic Bullying and Self-Esteem among Youths of Sexual Minorities in Quebec (Canada). Cien. Saude Colet..

[11] Gower A.L., Rider G.N., McMorris B.J., Eisenberg M.E. (2018). Bullying Victimization Among LGBTQ Youth: Critical Issues and Future Directions. Curr. Sex. Health Rep..

[12] Birkett M., Newcomb M.E., Mustanski B. (2015). Does It Get Better? A Longitudinal Analysis of Psychological Distress and Victimization in Lesbian, Gay, Bisexual, Transgender, and Questioning Youth. J. Adolesc. Health.

[13] Tucker J.S., Ewing B.A., Espelage D.L., Green H.D., de la Haye K., Pollard M.S. (2016). Longitudinal Associations of Homophobic Name-Calling Victimization With Psychological Distress and Alcohol Use During Adolescence. J. Adolesc. Health.

[14] Olweus D. (1993). Bullying at School: What We Know and What We Can Do.

[15] Rodríguez-Hidalgo A.J., Pantaleón Y., Calmaestra J. (2019). Psychological Predictors of Bullying in Adolescents from Pluricultural Schools: A Transnational Study in Spain and Ecuador. Front. Psychol..

[16] Rodríguez-Hidalgo A.J., Alcívar A., Herrera-López M. (2019). Traditional Bullying and Discriminatory Bullying around Special Educational Needs: Psychometric Properties of Two Instruments to Measure It. Int. J. Environ. Res. Public Health.

[17] Earnshaw V.A., Reisner S.L., Menino D.D., Poteat V.P., Bogart L.M., Barnes T.N., Schuster M.A. (2018). Stigma-Based Bullying Interventions: A Systematic Review. Dev. Rev..

[18] Rodríguez-Hidalgo A.J., Hurtado-Mellado A. (2019). Prevalence and Psychosocial Predictors of Homophobic Victimization among Adolescents. Int. J. Environ. Res. Public Health.

[19] Menesini E., Salmivalli C. (2017). Bullying in Schools: The State of Knowledge and Effective Interventions. Psychol. Health Med..

[20] Peter T., Taylor C., Chamberland L. (2015). A Queer Day in Canada: Examining Canadian High School Students ’ Experiences With School-Based Homophobia in Two Large-Scale Studies. J. Homosex..

[21] Rivers I., Gonzalez C., Nodin N., Peel E., Tyler A. (2018). LGBT People and Suicidality in Youth: A Qualitative Study of Perceptions of Risk and Protective Circumstances. Soc. Sci. Med..

[22] Gattamorta K., Quidley-Rodriguez N. (2017). Coming Out Experiences of Hispanic Sexual Minority Young Adults in South Florida. J. Homosex..

[23] Herrera-López M., Romera E., Ortega-Ruiz R. (2017). Bullying y Cyberbullying En Colombia; Coocurrencia En Adolescentes Escolarizados. Rev. Latinoam. Psicol..

[24] Rodríguez-Hidalgo A.J., Mero O., Solera E., Herrera-López M., Calmaestra J. (2020). Prevalence and Psychosocial Predictors of Cyberaggression and Cybervictimization in Adolescents: A Spain-Ecuador Transcultural Study on Cyberbullying. PLoS ONE.

[25] Rodríguez-Hidalgo A.J., Solera E., Calmaestra J. (2018). Psychological Predictors of Cyberbullying According to Ethnic-Cultural Origin in Adolescents: A National Study in Spain. J. Cross. Cult. Psychol..

[26] Ybarra M.L., Mitchell K.J., Palmer N.A., Reisner S.L. (2015). Online Social Support as a Buffer against Online and Offline Peer and Sexual Victimization among U.S. LGBT and Non-LGBT Youth. Child Abuse Negl..

[27] Espelage D.L., Basile K.C., De La Rue L., Hamburger M.E. (2015). Longitudinal Associations Among Bullying, Homophobic Teasing, and Sexual Violence Perpetration Among Middle School Students. J. Interpers. Violence.

[28] Humphrey T., Vaillancourt T. (2020). Longitudinal Relations between Bullying Perpetration, Sexual Harassment, Homophobic Taunting, and Dating Violence: Evidence of Heterotypic Continuity. J. Youth Adolesc..

[29] Rodríguez-Hidalgo A.J., Pincay A.A., Payán A.M., Herrera-López M., Ortega-Ruiz R. (2021). Los Predictores Psicosociales Del Bullying Discriminatorio Debido Al Estigma Ligado a Las Necesidades Educativas Especiales (NEE) y La Discapacidad. Psicol. Educ..

[30] Elipe P., Muñoz M., Del Rey R. (2018). Homophobic Bullying and Cyberbullying: Study of a Silenced Problem. J. Homosex..

[31] Siller L., Edwards K.M., Banyard V. (2020). Violence Typologies Among Youth: A Latent Class Analysis of Middle and High School Youth. J. Interpers. Violence.

[32] Cuadrado I., Fernández Antelo I., Martín-Mora Parra G. (2019). ¿Pueden Las Víctimas de Bullying Convertirse En Agresores Del Ciberespacio? Estudio En Población Adolescente. Eur. J. Investig. Health Psychol. Educ..

[33] Khong J.Z.N., Tan Y.R., Elliott J.M., Fung D.S.S., Sourander A., Ong S.H. (2020). Traditional Victims and Cybervictims: Prevalence, Overlap, and Association with Mental Health Among Adolescents in Singapore. School Ment. Health.

[34] Elipe P., Del Rey R., Columbus A.M. (2017). Homophobic Bullying: An Old Problem Only Recently Brought to Light. Advances in Psychology Research.

[35] Del Rey R., Elipe P., Ortega-Ruiz R. (2012). Bullying and Cyberbullying: Overlapping and Predictive Value of the Co-Occurrence. Psicothema.

[36] Ortega-Ruiz R., Del Rey R., Casas J.A. (2016). Evaluar El Bullying y El Cyberbullying Validación Española Del EBIP-Q y Del ECIP-Q. Psicol. Educ..

[37] Olweus D. (2012). Cyberbullying: An Overrated Phenomenon?. Eur. J. Dev. Psychol..

[38] Baruch-Dominguez R., Infante-Xibille C., Saloma-Zuñiga C.E. (2016). Homophobic Bullying in Mexico: Results of a National Survey Homophobic Bullying in Mexico: Results of a National. J. LGBT Youth.

[39] Hutzell K.L., Payne A.A. (2017). The Relationship between Bullying Victimization and School Avoidance: An Examination of Direct Associations, Protective Influences, and Aggravating Factors. J. Sch. Violence.

[40] Jones L.M., Mitchell K.J., Turner H.A., Ybarra M.L. (2018). Characteristics of Bias-Based Harassment Incidents Reported by a National Sample of U. S. Adolescents. J. Adolesc..

[41] Hidalgo-Rasmussen C.A., Ramírez-López G., Rajmil L., Skalicky A., Hidalgo-San Martín A. (2018). Bullying and Health-Related Quality of Life in Children and Adolescent Mexican Students. Cienc. e Saude Coletiva.

[42] Muñoz-Reyes J.A., Polo P., Valenzuela N., Guerra R., Anabalón K., Hidalgo-Rasmussen C., Turiégano E. (2018). Sexual Differences and Associations between Aggressiveness and Quality of Life in Late Adolescents. Curr. Psychol..

[43] Beckman L., Svensson M., Frisén A. (2016). Preference-Based Health-Related Quality of Life among Victims of Bullying. Qual. Life Res..

[44] Haraldstad K., Kvarme L.G., Christophersen K.A., Helseth S. (2019). Associations between Self-Efficacy, Bullying and Health-Related Quality of Life in a School Sample of Adolescents: A Cross-Sectional Study. BMC Public Health.

[45] Bogart L.M., Elliott M.N., Klein D.J., Tortolero S.R., Mrug S., Peskin M.F., Davies S.L., Schink E.T., Schuster M.A. (2014). Peer Victimization in Fifth Grade and Health in Tenth Grade. Pediatrics.

[46] Haraldstad K., Christophersen K.A., Eide H., Nativg G.K., Helseth S. (2011). Predictors of Health-Related Quality of Life in a Sample of Children and Adolescents: A School Survey. J. Clin. Nurs..

[47] Albaladejo-Blázquez N., Ferrer-Cascales R., Ruiz-Robledillo N., Sánchez-Sansegundo M., Fernández-Alcántara M., Delvecchio E., Arango-Lasprilla J.C. (2019). Health-Related Quality of Life and Mental Health of Adolescents Involved in School Bullying and Homophobic Verbal Content Bullying. Int. J. Environ. Res. Public Health.

[48] Diaz Herraiz E., Gutierrez R. (2017). The Health-Related Quality of Life of Students Involved in School Bullying. Int. J. Sch. Cogn. Psychol..

[49] Ravens-Sieberer U., Gosch A., Rajmil L., Erhart M., Bruil J., Power M., Duer W., Auquier P., Cloetta B., Czemy L. (2008). The KIDSCREEN-52 Quality of Life Measure for Children and Adolescents: Psychometric Results from a Cross-Cultural Survey in 13 European Countries. Value Health.

[50] González-Cabrera J., León-Mejía A., Beranuy M., Gutiérrez-Ortega M., Alvarez-Bardón A., Machimbarrena J.M. (2018). Relationship between Cyberbullying and Health-Related Quality of Life in a Sample of Children and Adolescents. Qual. Life Res..

[51] Earnshaw V.A., Bogart L.M., Poteat V.P., Reisner S.L., Schuster M.A. (2016). Bullying Among Lesbian, Gay, Bisexual, and Transgender Youth. Pediatr. Clin. North Am..

[52] Rivera-Osorio J.F., Arias-Gómez M.C. (2020). Acoso Escolar Contra Jóvenes LGBT e Implicaciones Desde Una Perspectiva de Salud. Rev. la Univ. Ind. Santander. Salud.

[53] Wang C.-C., Lin H.-C., Chen M.-H., Ko N.-Y., Chang Y.-P., Lin I.-M., Yen C.-F. (2018). Effects of Traditional and Cyber Homophobic Bullying in Childhood on Depression, Anxiety, and Physical Pain in Emerging Adulthood and the Moderating Effects of Social Support among Gay and Bisexual Men in Taiwan. Neuropsychiatr. Dis. Treat..

[54] Paul Poteat V., Espelage D.L., Koenig B.W. (2009). Willingness to Remain Friends and Attend School with Lesbian and Gay Peers: Relational Expressions of Prejudice among Heterosexual Youth. J. Youth Adolesc..

[55] Brighi A., Ortega R., Scheitauer H., Smith P.K., Tsormpatzoudis C., Barkoukis V., Del Rey R., Thompson J. European Bullying Intervention Project Questionnaire (EBIPQ), 2012.

[56] Del Rey R., Casas J.A., Ortega-Ruiz R., Schultze-Krumbholz A., Scheithauer H., Smith P., Thompson F., Barkoukis V., Tsorbatzoudis H., Brighi A. (2015). Structural Validation and Cross-Cultural Robustness of the European Cyberbullying Intervention Project Questionnaire. Comput. Human Behav..

[57] Rajmil L., Herdman M., Fernandez M.-J., Detmar S., Bruil J., Ravens-Sieberer U., Bullinger M., Simeoni M.-C., Auquier P. (2004). Generic Health-Related Quality of Life Instruments in Children and Adolescents: A Qualitative Analysis of Content. J. Adolesc. Health.

[58] Romeo K.E., Chico E., Darcangelo N., Bellinger L.B., Horn S.S. (2017). Youth’s Motivations for Using Homophobic and Misogynistic Language. J. LGBT Youth.

[59] Jadva V., Guasp A., Bradlow J.H., Bower-Brown S., Foley S. (2021). Predictors of Self-Harm and Suicide in LGBT Youth: The Role of Gender, Socio-Economic Status, Bullying and School Experience. J. Public Health.

[60] Hemphill S.A., Kotevski A., Tollit M., Smith R., Herrenkohl T.I., Toumbourou J.W., Catalano R.F. (2012). Longitudinal Predictors of Cyber and Traditional Bullying Perpetration in Australian Secondary School Students. J. Adolesc. Health.

[61] Juvonen J., Graham S. (2014). Bullying in Schools: The Power of Bullies and the Plight of Victims. Annu. Rev. Psychol..

[62] Hall W.J. (2018). Psychosocial Risk and Protective Factors for Depression Among Lesbian, Gay, Bisexual, and Queer Youth: A Systematic Review. J. Homosex..

[63] Chen Y.Y., Huang J.H. (2015). Precollege and In-College Bullying Experiences and Health-Related Quality of Life among College Students. Pediatrics.

[64] Sterzing P.R., Gibbs J.J., Gartner R.E., Goldbach J.T. (2018). Bullying Victimization Trajectories for Sexual Minority Adolescents: Stable Victims, Desisters, and Late-Onset Victims. J. Res. Adolesc..

